# A case report and literature review of possible multiple system atrophy–parkinsonian type with cholinergic deficiency

**DOI:** 10.1111/cns.14243

**Published:** 2023-04-30

**Authors:** Meng Wang, Yajuan Wang, Yuyuan Yang, Moxin Luan, Meixiang Zhong, Lulu Xu, Xueping Zheng

**Affiliations:** ^1^ Department of Geriatric Medicine The Affiliated Hospital of Qingdao University Qingdao China; ^2^ Department of Geriatric Medicine The Qingdao Eighth People's Hospital Qingdao China

Dear editor

Multiple system atrophy (MSA) is an adult‐onset, fatal neurodegenerative disease presenting with primarily cerebellar dysfunction (MSA‐C) or parkinsonian dysfunction (MSA‐P). Patients often show autonomic dysfunction in the early stages of the disease. The disease is rare and difficult to diagnose. Here, we report a case of a possible MSA‐P patient with cholinergic deficiency.

A 50‐year‐old woman was admitted to our department in January 2022 due to constipation for more than 10 years that had been aggravated since the last month. The timeline of the disease course, diagnosis, and treatment is presented in Figure 1. She developed urinary retention in the last month before admission. The patient was previously admitted to our hospital in December 2019 due to palpitations lasting for 2 months. One year later, in December 2020, the patient presented with right upper clumsiness and bradykinesia. She also revealed that she had been experiencing limb fidgeting and yelling in her sleep for years. In January 2021, she developed an involuntary limb tremor in her right arm, dragging and leaning to the right when walking, anhidrosis, and urinary retention. The patient's constipation started aggravating from then onward. In March 2021, she was admitted for the second time and diagnosed with parkinsonian syndrome. Levodopa/benserazide (100/25 mg TID) did not work as well in her symptom; an increase in the dose of the same to 200/50 mg TID resulted in transient partial alleviation.

**FIGURE 1:**
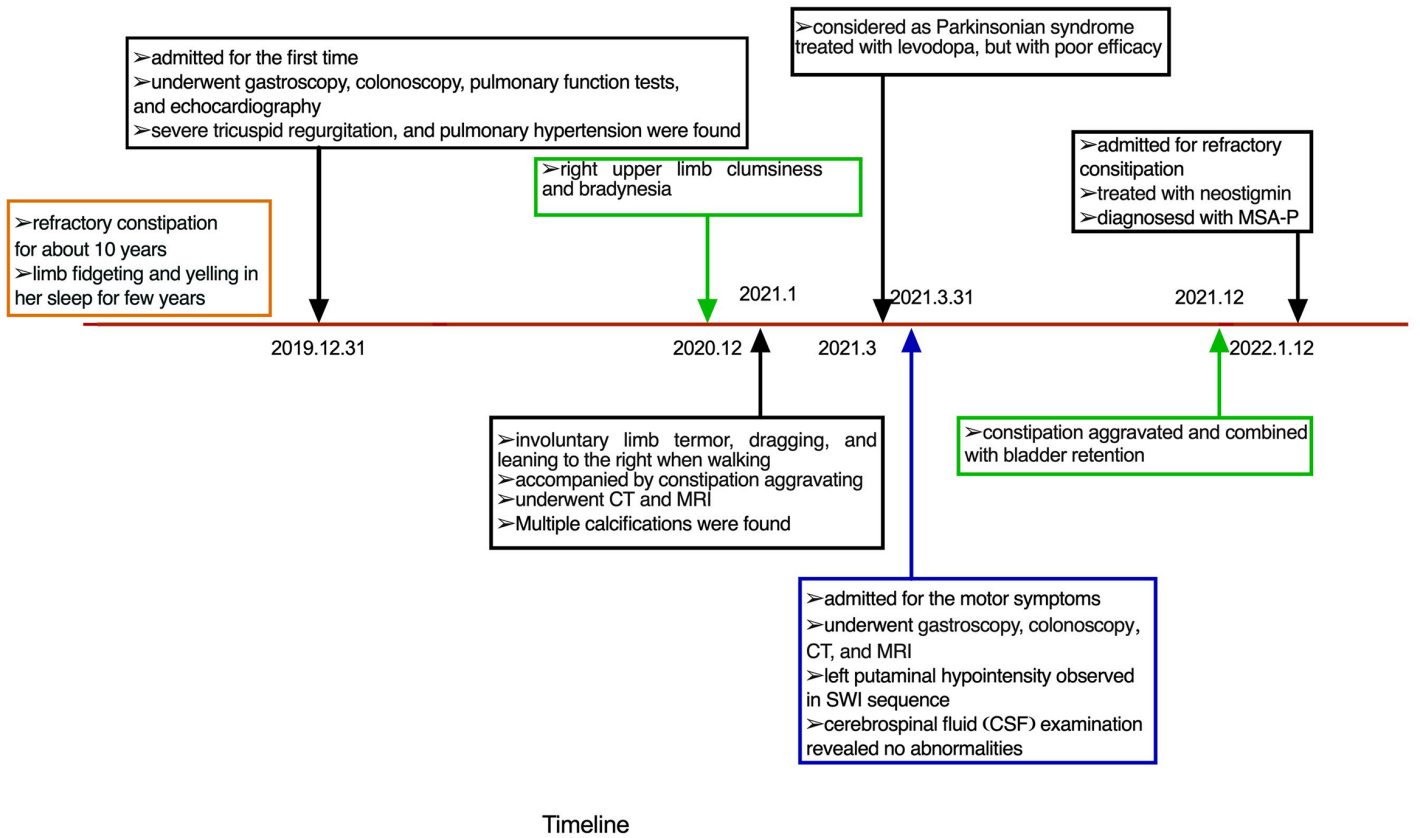
Timeline of the disease course, diagnosis, and treatment. A 50‐year‐old woman was admitted to our department in January 2022 due to constipation for more than 10 years that had been aggravated in the last month. She defecated once every 7–8 days; she was treated with glycerin enema and took lactulose for a long time, without evident improvement. Additionally, she developed urinary retention in the last month before admission. Urological ultrasound performed at a local hospital revealed right renal insufficiency, right hydronephrosis, and right renal calculus; thus, catheterization was performed. The patient was previously admitted to our hospital in December 2019 due to palpitations lasting for 2 months. Moderate tricuspid regurgitation and pulmonary arterial hypertension were observed on echocardiography, suggesting congenital heart disease. Chest CT showed multiple calcifications in the lungs. Gastroscopy revealed chronic non‐atrophic *Helicobacter pylori*‐positive gastritis. At that time, the patient's chronic constipation was considered to be related to pelvic floor muscle dysfunction and was treated with laxatives. One year later, in December 2020, the patient presented with right upper clumsiness and bradykinesia. She also revealed that she had been experiencing limb fidgeting and yelling in her sleep for years. In January 2021, she developed an involuntary limb tremor in her right arm, dragging and leaning to the right when walking, anhidrosis, and urinary retention. The patient's constipation started aggravating from then onward. In March 2021, she was admitted for the second time and diagnosed with parkinsonian syndrome. She was treated with levodopa/benserazide (100/25 mg TID) with poor efficacy; an increase in the dose of the same to 200/50 mg TID resulted in transient partial alleviation.

No obvious abnormalities were found on general physical examination, except for a grade 3/6 holosystolic murmur that was audible over the tricuspid valve area. Neurological examination showed hypertonia in the right extremity and normal muscular tonicity in the left extremity, along with normal muscle strength in all four limbs. Tendon reflex was brisk, and abdominal reflex was absent. Rossolimo signs were positive bilaterally, and right plantar response was absent. Whole‐genome sequencing showed no clear pathogenicity variation associated with a specific disease phenotype for renal atrophy, multiple calcifications, pulmonary arterial hypertension, tricuspid regurgitation, MSA, and early‐onset PD.

The patient was suspected of having intestinal obstruction because of her refractory constipation. As the patient had anal exhaust and abdominal CT showed no air‐fluid levels, intestinal pseudo‐obstruction was considered. Thus, neostigmine was administered at a dose of 0.1 g. She defecated well after approximately 10 min but developed severe abdominal pain, sweating, and tremor, which are signs of cholinergic deficiency. Having symptoms of parkinsonism, persistent refractory constipation, and unexplained incomplete bladder emptying, the patient was diagnosed as possible MSA‐P. At the 2‐week follow‐up, the patient reported no further symptoms of pseudo‐obstruction‐induced constipation after discharge. At the 2‐month follow‐up, her Parkinson's symptoms aggravated after discharge, and she was prescribed levodopa/benserazide (200/50 mg TID) and pramipexole (0.125 mg BID). The patient had partial alleviation for only a short time.

In MSA, patients usually present with intact postganglionic noradrenergic fibers.^1^ Accordingly, cases of MSA combined with cholinergic deficiency have rarely been reported. We reviewed all cases of MSA associated with cholinergic deficiency and we evaluated the different patterns of presentation and age at onset (Table 1). Our analysis revealed that all patients had parkinsonian symptoms and pyramidal signs. The presentation of cholinergic deficiency differed among these cases, exhibiting central sleep apnea, nicotinic, or muscarinic symptoms. However, none of these patients showed the typical MSA neuroimaging findings of “hot cross bun” sign or dilatation of the fourth ventricle (Figure 2A,B).^2^ Only one case had mild cerebellar atrophy, and our patient showed left putaminal hypointensity (Figure 2C,D). These patients had severe and early autonomic symptoms, while the typical MSA neuroimaging findings cannot be detected in the early stage. Whether the symptoms of cholinergic deficiency could contribute to the early diagnosis of MSA needs further study.

**TABLE 1:** Summary of the clinical features of cases with cholinergic deficiency.

No./study	Gender/age	diagnosis	Presentation of motor disorder	Autonomic failure presentation	Cholinergic deficiency presentation	Neuroimaging	Other tests	Neurological examination	Follow‐up
Graham et al. (1969)^8^	Male/60	Shy‐Drager syndrome	Unsteadiness of gait	Urinary hesitancy, dribbling, nocturnal incontinence, sexual impotence, orthostatic hypotension	Unsteadiness worsened and made his legs feel “wooden” by smoking	Air encephalogram showing normal posterior fossa anatomy, slight ventricular dilatation, and a slight excess of air in the cerebral cortical sulci	A normal EEG, normal cerebrospinal fluid, normal chest, and skull radiographed	Reflexes hyperactive, brisk finger jerks, left lower limbs ataxic, right plantar response absent	Died of urinary and chest infections at 62
Colosimo et al. (2012)^9^	Male/64	MSA‐C	Gait and balance difficulties, dysarthria, and hand tremor	Bladder retention, fecal incontinence, sexual dysfunction, sleep apnea	Motor disorders worsened by smoking a cigarette	Routine brain magnetic resonance imaging result was normal	Orthostatic hypotension with severely impaired sympathetic and vagal functions	Ataxic in both upper limbs, truncal ataxia evident, tendon reflexes brisk	Not mentioned
Cormican et al. (2004)^10^	Male/61	Probable MSA‐C	Slurred speech, ataxia	Urinary incontinence, loss of early morning erections	Central sleep apnea may be due to the depletion of cholinergic neurons	MRI of brain and brainstem revealed mild cerebellar atrophy and nonspecific white matter lesions, otherwise normal	Electroencephalogram revealed nonspecific abnormalities, no epileptiform discharges	Symptoms attributed to hypoxia and hypercapnia, eventually resolved	Re‐admitted for increasing dependency on his carers at 65
This study	Female/50	Possible MSA‐P	Involuntary shaking of limbs, dragging	Anhidrosis, urine retention, refractory constipation	After Neostigmine treatment, defecated, abdominal pain, sweating	Cerebral CT scan showed multiple calcifications in frontal lobe, left putaminal hypointensity in SWI sequence, otherwise normal	Severe tricuspid regurgitation, multiple abdominal calcifications cerebrospinal fluid examination revealed no abnormalities.	Abdominal reflex absent, right lower limb hypertonia, right plantar response absent, positive Rossolimo	Follow‐up

**FIGURE 2:**
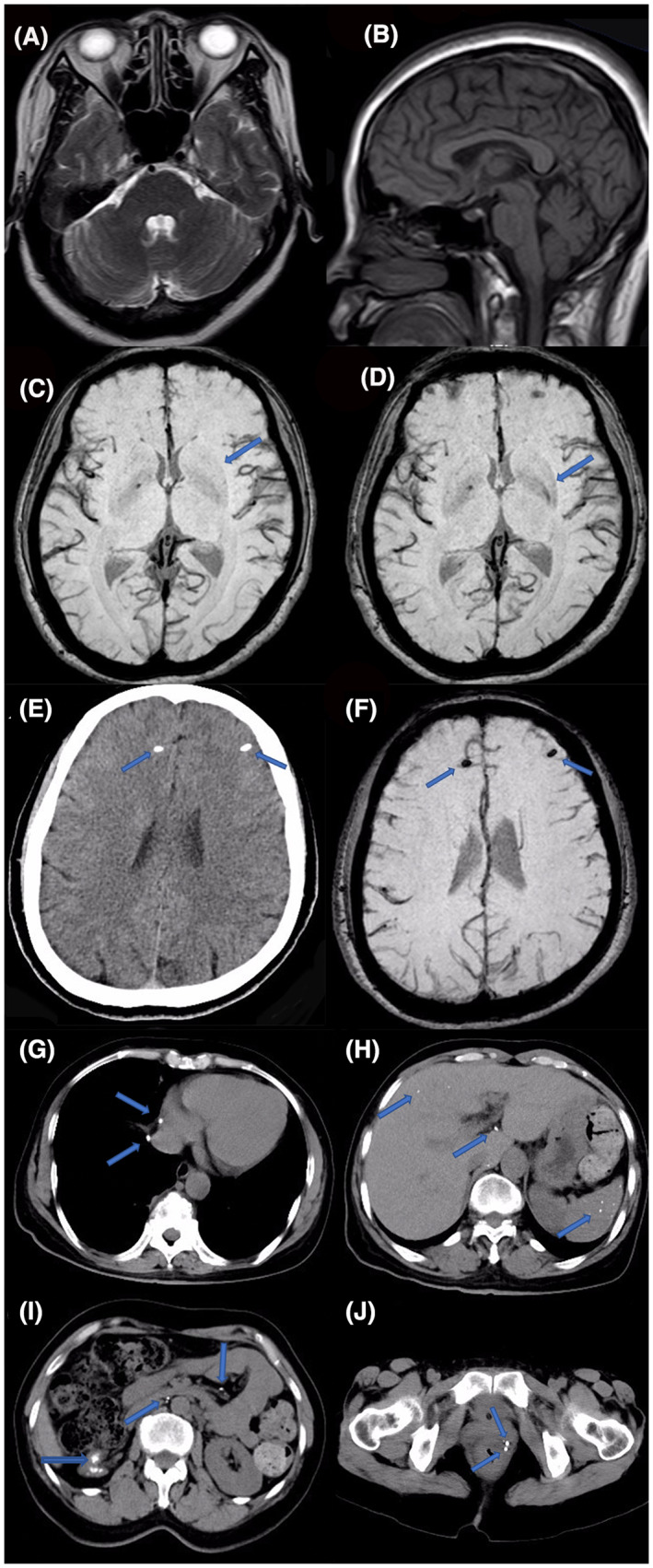
Brain computed tomography (CT) and magnetic resonance imaging (MRI) findings; brain susceptibility weighted imaging (SWI) sequence of putamen; chest, abdominal, and pelvic CT findings. Axial and sagittal brain MR of the patient showed no abnormality. Axial brain MRI showed no “Hot cross bun” (A) and sagittal brain MRI showed no infratentorial atrophy (B); no obvious putaminal signal changes were observed before the onset of symptom of MSA‐P in 2020 (C, blue arrow). The left putaminal hypointensity observed in 2021 (D, blue arrow). Multiple calcifications in frontal lobe on cerebral CT and SWI sequence, Multiple calcifications (blue arrows) in frontal lobe on cerebral CT (E), and the multiple calcifications (blue arrows) in the same place on SWI sequence (F). Multiple calcifications (blue arrows) in inferior vena (G), liver, spleen (H), abdominal cavity, right kidney (I), and bladder (J).

The most prominent symptom of autonomic disorder in our case was refractory constipation. Cortical cholinergic denervation resulting from degeneration of the nucleus basalis of Meynert is a primary contributor of the cognitive impairment and neuropsychiatric symptoms often be associated with Lewy body diseases. Neurodegeneration in the nucleus basalis of Meynert and striatum may affect noradrenergic, dopaminergic, serotonergic, and cholinergic neurons.^3^ Gilman et al.^4^ found that acetylcholinesterase activity was reduced in the MSA‐P striatum, which further confirmed the assumption. Additionally, α‐synuclein accumulation was reported in the enteric nervous system in MSA^5^ and might contribute to gastrointestinal dysfunction. Thus, the cholinergic deficiency in MSA may be associated with α‐synuclein accumulation in the striatum, and α‐synuclein accumulation in the enteric nervous system may further aggravate constipation in MSA.

In addition, another interesting finding for our patient was that her imaging findings showed multiple calcifications in the frontal lobe, liver, spleen, abdominal cavity, and both kidneys (Figure 2E–J). Furthermore, as no literature to support there is an association between calcification and cholinergic deficiency, we speculate that the coexistence of multiple calcifications and cholinergic insufficiency may happen accidentally. This case remains controversial. First, the patient had no symptoms of orthostatic hypotension and exhibited asymmetrical dyskinesia, which was not consistent with the typical clinical manifestations of MSA. Second, the relationship between MSA and her previous constipation could not be confirmed because of her history of more than 10 years of constipation. Second, the mean survival of such patients from the onset of symptoms is 6–10 years.^6^, ^7^ Thus, long‐term follow‐up is needed, and the levodopa dose should be further increased to evaluate the treatment response. Fourth, the patient had multiple unexplained calcifications, renal atrophy, severe tricuspid regurgitation, and pulmonary hypertension. None of these abnormalities could be well explained. Finally, the definite diagnosis of MSA requires pathological demonstration. Due to the lack of neuropathology, this patient could not be diagnosed as definite MSA‐P. Therefore, there are still more questions than answers about the diagnosis and treatment of this patient, long‐term follow‐up is still needed. And, in patients with severe autonomic symptoms, especially cholinergic deficiency, the diagnosis of MSA should not be ignored clinically.

## FUNDING INFORMATION

This work was supported by the Affiliated Hospital of Qingdao University (QDFY) (X202101032), Qingdao University of Science and Technology (QUST) grants (WST 2021020), and Health Commission of Qingdao (2022‐WJZD184).

## CONFLICT OF INTEREST STATEMENT

The authors declare that they have no conflicts of interest.

## Data Availability

All data associated with this study are present in the paper.
